# The Dark Side of the Epitranscriptome: Chemical Modifications in Long Non-Coding RNAs

**DOI:** 10.3390/ijms18112387

**Published:** 2017-11-10

**Authors:** Roland Jacob, Sindy Zander, Tony Gutschner

**Affiliations:** Faculty of Medicine, Martin-Luther-University Halle-Wittenberg, 06120 Halle (Saale), Germany; roland.jacob@uk-halle.de (R.J.); sindy.zander2@uk-halle.de (S.Z.)

**Keywords:** cancer, epitranscriptomics, lncRNA, noncoding RNA, 5-methylcytosine, m^5^C, *N*^6^-methyladenosine, m^6^A, pseudouridine

## Abstract

The broad application of next-generation sequencing technologies in conjunction with improved bioinformatics has helped to illuminate the complexity of the transcriptome, both in terms of quantity and variety. In humans, 70–90% of the genome is transcribed, but only ~2% carries the blueprint for proteins. Hence, there is a huge class of non-translated transcripts, called long non-coding RNAs (lncRNAs), which have received much attention in the past decade. Several studies have shown that lncRNAs are involved in a plethora of cellular signaling pathways and actively regulate gene expression via a broad selection of molecular mechanisms. Only recently, sequencing-based, transcriptome-wide studies have characterized different types of post-transcriptional chemical modifications of RNAs. These modifications have been shown to affect the fate of RNA and further expand the variety of the transcriptome. However, our understanding of their biological function, especially in the context of lncRNAs, is still in its infancy. In this review, we will focus on three epitranscriptomic marks, namely pseudouridine (Ψ), *N*^6^-methyladenosine (m^6^A) and 5-methylcytosine (m^5^C). We will introduce writers, readers, and erasers of these modifications, and we will present methods for their detection. Finally, we will provide insights into the distribution and function of these chemical modifications in selected, cancer-related lncRNAs.

## 1. Introduction

Non-genomically encoded modifications of macromolecules, ranging further than simple changes in the sequence of the single building blocks, play important roles in nearly all cellular processes. The need to regulate activities and abundances of working components led to mechanisms involving several layers of control. For example, post-translational modifications of proteins, like phosphorylation, acetylation, ubiquitination, glycosylation and methylation are well-known modifications that control the fate of proteins [1].

The first modified nucleotide in DNA was discovered in 1948 [2]. In the following decades, the research field of epigenetics evolved, before the term “epigenetics” was eventually coined in the 1990s. It has been redefined more than once since then [3]. Today we know plenty about the processes of imprinting, gene silencing, X-chromosome inactivation and the function of epigenetics in cancer development. Nevertheless, there is a plethora of information still to be unearthed.

In contrast to DNA and proteins, RNA was neglected for a long time and thought to be just an intermediary component on the way from the information stored inside the DNA double helix to the readily synthesized proteins that are to fulfill all important tasks inside the cell. This view changed in the 1980s, when catalytic functions of RNA molecules were brought to light [4]. Only then the field of non-coding RNA (ncRNA) came into being and slowly began to evolve. More and more classes of RNAs were described, possessing important functions while not coding for a peptide chain [5]. Surprisingly, it was revealed that a large fraction (70–90%) of the human genome is transcribed into RNA. If one takes into account that only 1–3% of the transcriptome carries the blueprint for the synthesis of proteins, it leaves us with the question whether or not the remaining non-coding transcripts are just “trash” [6,7].

NcRNAs are somewhat arbitrarily divided into two classes depending on their size: (1) small ncRNAs (<200 nucleotides (nt)); and (2) long ncRNAs (lncRNAs). Multiple types of small ncRNA (microRNAs (miRNAs), small interfering RNAs (siRNAs) and PIWI-interacting RNAs (piRNAs)) have been studied extensively, especially their role in development and carcinogenesis [8,9,10,11,12,13].

The group of long ncRNAs is highly heterogeneous and its members have an extensive variability in their cellular effects as well as their molecular influences. They are characterized by the lack of a functional open reading frame, meaning they encompass less than 100 amino acids [14,15,16,17]. It is their heterogeneity, which allows them to cover a broad spectrum of molecular and cellular functions by implementing different modes of action [18,19,20,21,22,23,24,25]. Of note, a recent analysis identified a consensus human transcriptome of 91,013 expressed, polyadenylated genes. Importantly, 58,648 genes (~68%) were classified as lncRNAs [26]. The lncRNAdb is a database comprising the growing number of functionally annotated lncRNAs [27].

In 1951, pseudouridine (Ψ), the first modification of a RNA base, was discovered [28], only shortly after the description of 5-methylcytosine (“epicytosine”) in DNA [2,29]. With time, more and more nucleotide modifications were described. Today, over 150 modifications are known and several online databases are keeping track of the progress on this front [30,31,32].

For years, research on RNA modifications focused mainly on transfer RNAs (tRNAs) as a result of their relative abundance and their small size, with ribosomal RNA (rRNA) following after technological advances in sequencing methodology were made [33,34,35,36]. Only after the emergence of next-generation sequencing (NGS) technology in the last couple of years, it was feasible to shift the scope of research towards “transcriptome-wide” modification studies. Nearly all experimental designs involve an enrichment step for polyadenylated (polyA) RNA or some other kind of selection step. Unsurprisingly, messenger RNAs (mRNAs) that make up only a small fraction of the transcribed RNA population are at the center of attention [37]. Today it is evident that RNA modifications are more prevalent and chemically diverse than their DNA counterparts [31]. They are highly dynamic and at least some are reversible, which makes them a critical component of the post-transcriptional gene regulatory landscape. It is becoming clear that RNA modifications and alterations of the RNA modification machinery can have detrimental effects in human disease [38].

**Figure 1:**
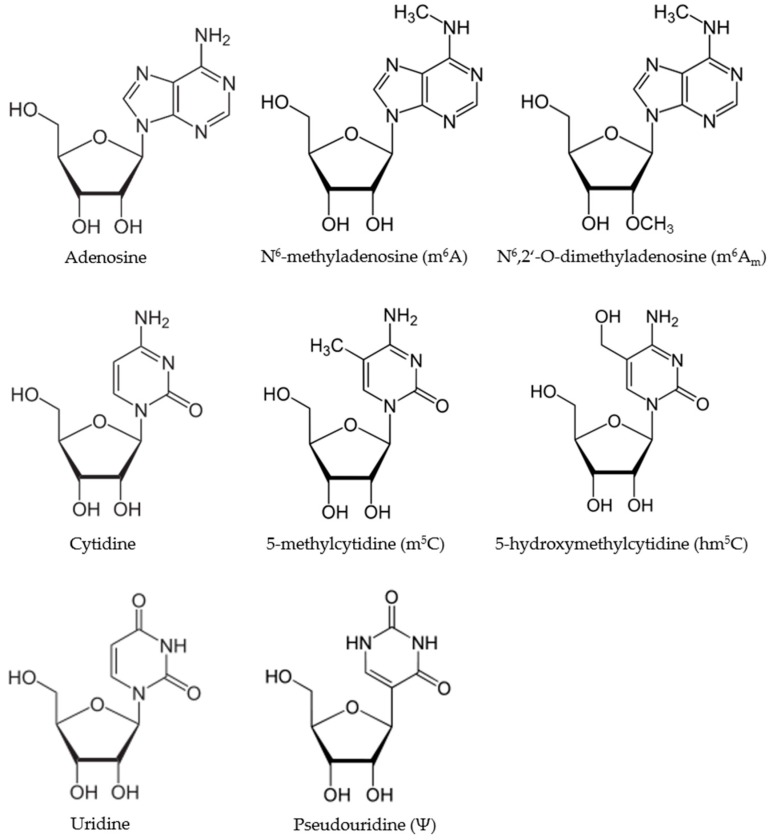
Selected chemical modifications present in RNA.

This review will focus on the three most abundant RNA modifications, namely, pseudouridine (Ψ), *N*^6^-methyladenosine (m^6^A) and 5-methylcytosine (m^5^C) (Figure 1). After describing each modification, including the known interacting proteins in greater detail, and highlighting the detection methods for each, we will turn our focus on selected examples of cancer-related lncRNAs that have recently been shown to be part of the emerging epitranscriptome.

## 2. Discovery and Function of RNA Modifications

Post-transcriptional modifications of RNA molecules have been known for nearly 70 years and about 150 epitranscriptomic marks have been described in the last decades. Chromatographic methods were used in early studies and they are still a very valuable tool for detection today. They remain the gold standard, especially for quantification of RNA modifications. However, reliable transcriptome-wide mapping of the most prevalent alterations with the help of next-generation sequencing technology is the ultimate goal today.

In this paragraph, we will briefly summarize our current knowledge about the three most widespread RNA modifications, namely, pseudouridine (Ψ), *N*^6^-methyladenosine (m^6^A) and 5-methylcytosine (m^5^C), their interacting proteins and connections to disease states. Moreover, we will introduce current high-throughput detection methods of the aforementioned modifications and briefly discuss their benefits and limitations. In Table 1, we provide an overview of the currently known proteins involved in writing, reading and erasing the three epitranscriptomic marks.

### 2.1. Pseudouridine

Overall, 5-ribosyluracil (pseudouridine, Ψ) is the most abundant RNA modification, first described in 1951 and found in several classes of RNA, i.e., tRNA, rRNA, small nuclear RNA (snRNA), small nucleolar RNA (snoRNA), mRNA and ncRNA [28]. It is an isomer of the conventional RNA nucleoside uridine (see Figure 1). As a result of its high abundance, Ψ was even termed “the fifth nucleotide” [41,73]. Almost all tRNA molecules possess at least one Ψ residue and the TΨC loop is a characteristic feature of tRNAs.

Incorporated Ψ nucleosides enhance RNA’s ability for base stacking and make the sugar-phosphate backbone more rigid [74,75]. Ψ engages in classical Watson–Crick base pairing with adenosine like its non-modified isomer uridine, though its pairing with all other four bases is stronger than uridine’s. Interestingly, the conversion of uridine to Ψ in translation termination codons was able to suppress translation termination in yeast where pseudouridine-containing stop codons guided the incorporation of selected amino acids [76]. Importantly, altered Ψ distribution patterns in mRNAs and in ncRNAs could be observed in yeast and human cells after stress application [77]. This demonstrates how RNA modifications can expand the genetic code and permit more flexibility to adapt to environmental factors.

In humans, 13 proteins have been identified that contain a pseudouridine synthase domain. These so called pseudouridine synthases (PUS) fall into one of two categories: RNA-dependent or RNA-independent. PUS from the first category rely on other small RNAs that guide these enzymes to their respective target RNAs. In contrast, PUS from the latter category can fulfill their catalytic duty without these adaptor RNAs. Dyskerin, for example, associates with H/ACA snoRNAs, while PUS1 belongs to the snoRNA-independent group [73].

Very recently, it was shown that TruB pseudouridine synthase family member 1 (TRUB1), which is also known as PUS4, and PUS7 combine for about 60% of all reproducibly detected Ψ sites in mRNA [44]. Moreover, a consensus motif (GUUCNANNC) for pseudouridylation by TRUB1 could be identified. While the function of TRUB2-dependent pseudouridylation of mRNA remains an open question, it could be shown that TRUB1 can localize to the nuclear and cytoplasmic compartments. However, its catalytic activity was suggested to be restricted to the nucleus [44]. In contrast, several other pseudouridine synthases (PUS1, pseudouridylate synthase-like 1 (PUSL1), TRUB2, RNA pseudouridylate synthase domain containing 3 (RPUSD3) and RPUSD4) have been predicted or proven to be localized, at least partially, to mitochondria [46,78]. Consequently, multiple mitochondrial RNAs (mtRNAs) are modified by PUS enzymes, e.g., mt-tRNA (RPUSD4 and PUS1), mt-rRNA (RPUSD4) and mt-mRNA (TRUB2 and RPUSD3) [46,78,79]. Intriguingly, deregulation of snoRNAs and mutations in pseudouridine synthases are associated with different diseases like lung cancer, mitochondrial myopathy, sideroblastic anaemia, and dyskeratosis congenita [47,80,81].

Until today, specific reader or eraser proteins for Ψ have not been found. The reason for the absence of an eraser protein could be the fact that the formed C–C bond between the base and the sugar (Ψ) is significantly more inert than the C–N bond (uridine). The Ψ formation could, therefore, be irreversible [74]. Hence, pseudouridylation is likely “read” by structural changes of the RNA molecule itself, which originate from the different properties of Ψ compared to uridine. This could affect the stability of RNA molecules and their interactions with proteins without the need of mediating proteins that specifically read the Ψ residues. Structural functions within RNA molecules and altered base pairing properties of pseudouridine have been described [73,76,82].

#### Ψ Detection Methods

High-throughput and site-specific mapping of Ψ in RNA relies on the unique reaction of Ψ with *N*-cyclohexyl-*N*′-(2-morpholinoethyl)carbodiimide metho-p-toluenesulfonate (CMCT) and the downstream application of next-generation sequencing [83,84]. Pseudo-seq, Ψ-seq and PSI-seq are all based on this workflow [77,85,86]. Extracted RNA is fragmented and treated with CMCT, which forms a covalent bond with Ψ, U and G residues. However, only the Ψ-CMC product is stable under alkaline conditions whereas U- and G-reaction products are getting hydrolyzed. Subsequently, the CMC-modified RNA is reverse transcribed. Importantly, reverse transcription will terminate one nucleotide 3′ to pseudouridylated sites due to the bulky (CMC-) group attached to Ψ residues. Hence, next-generation sequencing of cDNA libraries constructed with or without CMCT treatment allows one to map Ψ positions in RNA transcripts by calculating stop rate differences between these two samples.

A related, yet more sensitive method, called CeU-Seq, was recently developed [87]. Here, a derivative of CMCT, to which biotin can be added via click chemistry, is used to modify Ψ residues within RNA molecules. Subsequent pull-down of biotin-labeled transcripts with streptavidin beads leads to an enrichment of modified RNA molecules over non-modified ones. This results in a better signal-to-noise ratio and improves detection of Ψ-modified RNA transcripts of low abundance.

Next to these sequencing-based methods, alternative strategies have been introduced or are currently under development to map pseudouridine in RNA. For example, site-specific cleavage and radioactive-labeling followed by ligation-assisted extraction and thin-layer chromatography (SCARLET) is a low-throughput method to validate different RNA base modifications, e.g., Ψ, and to determine the stoichiometry at individual nucleotide positions [87,88]. SCARLET is described in greater detail in the m^6^A detection section below.

Analyzing Ψ modifications in RNA by mass spectrometry is another, rather challenging approach, because there is no mass difference between uridine and Ψ [89]. Accordingly, there is a need for chemical labels that can be introduced, either directly into the cells by addition to the growth medium or through chemical reaction of the isolated RNA ex vivo. Hence, there are no high-throughput methods yet, but advances in the field (even towards label-free approaches) are continuously made [90].

### 2.2. N^6^-Methyladenosine

*N*^6^-methyladenosine (m^6^A) was first discovered in 1974 [91,92]. It is found in snoRNAs, tRNAs, rRNAs and other ncRNAs, and is the most widespread base modification of mRNA. It accounts for 0.2–0.6% of all adenosines in mammalian mRNA with about three sites per transcript [93]. There are two slightly differing consensus motifs proposed in which m^6^A occurs: RRACH [94,95] and DRACH [96] (with D = G, A, or U; R = G or A; and H = C, A, or U). However, the distribution of m^6^A in mRNA is not random, but follows a certain pattern; it is often located near stop codons and in the 3′-untranslated region (UTR) suggesting a regulatory role in cellular processes [57,97]. Indeed, m^6^A modifications have been shown to play important roles in RNA stability (mRNA and ncRNA) [56,98], mRNA translation [59,99,100], secondary structure formation (mRNA and lncRNA) [61,101,102], alternative splicing, and polyadenylation [93,103] as well as subcellular RNA location [62,104]. Very recently, a novel role for m^6^A in the UV-induced DNA damage response pathways was reported [105]. Importantly, the levels of m^6^A in mRNA are highly dynamic and the modification is reversible. In fact, several m^6^A writer, reader, and eraser proteins have been identified [57,97,106]. This reinforces the idea that m^6^A modifications serve important functions and might be involved in cell signaling networks.

#### 2.2.1. m^6^A Writers

The m^6^A formation is catalyzed inside the nucleus by the m^6^A writer complex, which consists of the enzymatically active methyltransferase-like 3 (METTL3) protein and several interacting proteins [48]. Known interaction partners of METTL3 are: (a) methyltransferase-like 14 (METTL14); (b) Wilms’ tumor 1-associating protein (WTAP); (c) KIAA1429, also called vir like m^6^A methyltransferase associated (VIRMA); (d) RNA-binding motif protein 15 (RBM15), and; (e) RBM15B. METTL3 possesses a catalytically active methyltransferase domain and it is the principal m^6^A forming enzyme in polyadenylated mRNA, but it does not methylate rRNA [107]. METTL14, on the other hand, has a degenerated active site and it is not catalytically active in the heterodimer with METTL3 [108]. It binds to substrate RNA and forms extensive contacts with METTL3 whose enzymatic activity is enhanced by this molecular interaction [107,109]. Hence, METTL14 acts as a RNA adaptor protein, which greatly enhances the methyltransferase activity of the m^6^A writer complex. Knockdown of METTL3 or METTL4 in glioblastoma stem-like cells (GSCs) dramatically increased their growth and self-renewal. In addition, this depletion substantially increased GSC-initiated tumor progression [110].

WTAP is a crucial component of the writer complex [49,51]. One of its functions is to localize the METTL3-METTL14-complex to nuclear speckles [50].

KIAA1429 is associated with the writer complex and its depletion led to a decrease in m^6^A abundance in RNA [51]. However, its molecular function is still obscure.

RBM15 and its paralog RBM15B are components of the methyltransferase complex and they interact with METTL3 in a WTAP-dependent manner [53,54]. RBM15/15B use their RNA-binding domains to enable the binding of the writer complex to specific mRNAs and even specific sites within these. The lncRNA *X*-inactive specific transcript (*XIST*), for instance, is a target of RBM15/15B directed methylation [54].

Recently, METTL16 was described as a methyltransferase fulfilling its functions independently of the m^6^A writer complex surrounding METTL3 [55]. It is a conserved U6 snRNA methyltransferase, and it has evolved an additional function in vertebrates to control *S*-Adenosyl methionine (SAM) homeostasis by differentially methylating a hairpin structure inside the methionine adenosyltransferase 2A (MAT2A) mRNA thereby modulating alternative splicing [55].

#### 2.2.2. m^6^A Readers

The m^6^A modifications in RNA transcripts are predominantly read by the eukaryotic initiation factor 3 (eIF3) or by proteins that contain a YTH (YT521-B homology) domain. There are additional RNA-binding proteins (RBPs) associating with m^6^A, which are not seen as classical m^6^A binders.

DF1, DF2 and DF3 belong to the YT521-B homology domain family (YTHDF) and represent one group of cytoplasmic m^6^A reader proteins [57]. DF1 is involved in modulating translation efficiency whereas DF2 is proposed to have a function in mRNA stability [56,99]. Additionally, it was reported that DF2 can localize to the nucleus after stress induction where it promotes cap-independent translation initiation [100].

A second group of m^6^A reader proteins are YTH domain-containing proteins (YTHDCs). YTHDC1 is a nuclear enriched protein that binds to protein-coding and non-coding transcripts. It is the major reader of nuclear m^6^A modifications [106]. YTHDC1 is a mediator of the X-chromosome silencing effect of *XIST* and was characterized as a regulator of mRNA splicing events [54,103]. YTHDC2’s functions are poorly defined. It is located inside the nucleus as well as in the cytoplasm and was shown to bind to select m^6^A sites in ncRNAs [54]. Tanabe et al. linked upregulated expression of YTHDC2 to metastasis in colon cancer [111].

Another important m^6^A binding protein is eIF3. In fact, adenosine methylation is a major mechanism by which eIF3 is recruited to mRNAs. After binding to m^6^A in the 5′-UTR, translation is initiated by eIF3 in a 5′-cap- and eukaryotic initiation factor 4E (eIF4E)-independent manner [59]. These findings suggest an alternative way of translation initiation mediated by m^6^A modifications in 5′-UTRs of mRNAs when eIF4-dependent initiation is hindered by specific cell states.

Finally, heterogeneous nuclear ribonucleoprotein C (hnRNP C) and hnRNP A2/B1 belong to the group of proteins with reported binding to m^6^A after changes in local and secondary structure of mRNA and lncRNA [61]. Binding of these proteins to m^6^A-containing transcripts has been shown to affect alternative splicing as well as miRNA biogenesis [60,61]. Interestingly, the well-characterized, AU-rich element (ARE) and poly(A)-binding protein human antigen R (HuR) preferentially binds to sequences that lack m^6^A modifications, and loss of m^6^A methylation enhances HuR binding, which increases target RNA stability [98]. Further research will be necessary to illuminate the connection between the binding of those proteins and cellular processes.

#### 2.2.3. m^6^A Erasers

The nuclear α-ketoglutarate-dependent dioxygenase alkB homolog 5 (ALKBH5) protein was recently identified as a RNA demethylating enzyme [62]. *Alkbh5*-deficient mice show defects in spermatogenesis, but are otherwise viable indicating that Alkbh5 demethylase activity is not strictly required during development [62]. Redundant demethylation pathways might be in place as well. In contrast, ALKBH5-mediated demethylation of m^6^A transcripts seems to be crucial in certain cancers. For example, Zhang et al. could show that ALKBH5 protein levels are elevated in GSCs and its expression is a negative prognostic factor for glioblastoma (GBM) patients [112]. Furthermore, the authors reported that ALKBH5 demethylates nascent *FOXM1* transcripts which results in enhanced FOXM1 expression. Interestingly, *FOXM1-AS*, a nuclear lncRNA, facilitates the interaction between ALKBH5 and nascent *FOXM1* transcripts. Depletion of ALKBH5 and *FOXM1-AS* disrupted GSC tumorigenesis through the FOXM1 axis [112]. In addition to its role in GBM, ALKBH5 expression is reported to be induced by hypoxia in breast cancer cells. Knockdown of ALKBH5 expression in MDA-MB-231 human breast cancer cells significantly reduced their capacity for tumor initiation as a result of reduced numbers of breast cancer stem cells (BCSCs) [113].

Very recent publications spawned conflicting data concerning the fat mass- and obesity-associated protein (FTO), a member of the AlkB-related dioxygenase family, which was originally described as an eraser of m^6^A modifications in RNA [63]. Recently, Mauer et al. reported that FTO acts as an eraser for the closely related *N*^6^, 2′-*O*-dimethyladenosine (m^6^A_m_) modification, which was co-detected with m^6^A in previous studies [114]. Their refined detection technique made it possible to differentiate between these two modifications, allowing a more detailed examination of FTO’s substrate spectrum. As a result, the authors could show that m^6^A is not the preferred target for FTO in vivo and they concluded that FTO is the eraser protein for m^6^A_m_.

In contrast, other reports showed a substantial increase in mRNA m^6^A levels in GSCs treated with the FTO inhibitor MA2, which suppressed GSC-initiated tumorigenesis and prolonged the lifespan of GSC-engrafted mice [110]. Another recent publication shed light on the role of FTO in acute myeloid leukemia (AML). Li et al. indicated that FTO, as a m^6^A demethylase, plays a critical oncogenic role in AML [115]. FTO is highly expressed in certain AMLs and it enhances oncogene-mediated cell transformation and leukemogenesis. It does so by reducing m^6^A levels in specific mRNA transcripts [115].

#### 2.2.4. m^6^A Detection

First high-throughput m^6^A mapping strategies were based on the immunoprecipitation of modified RNA molecules using m^6^A-specific antibodies coupled to the subsequent application of NGS technologies (m^6^A-seq [57], MeRIP-Seq [97]). Here, isolated and poly(A)-enriched RNA is fragmented to about 100 nt long fragments, which are immunoprecipitated with m^6^A-specific antibodies. Thereafter, cDNA libraries are constructed from m^6^A-containing samples as well as non-immunoprecipitated input control samples and subjected to sequencing. NGS reads are then mapped to the reference genome. Fragments containing m^6^A will be enriched and provide more reads. Through algorithm-based position calling, m^6^A positions can be determined. However, both methods provide a rather low resolution (100–200 nt), because peaks can be broad and identification of a single modified adenosine residue can be difficult. The same is true for m^6^A residues that lie in close proximity to each other. Therefore, true identification of specific m^6^A residues on a transcriptome-wide level is not possible with m^6^A-seq or MeRIP-Seq. Another drawback of these methods is the specificity of the available antibodies. These recognize m^6^A as well as *N*^6^,2′-*O*-dimethyladenosine (m^6^A_m_), which both contain the 6-methyladenine base. Therefore, it is not possible to distinguish between the prevalent m^6^A and m^6^A_m_, which is found close to the 5′-cap of mRNAs [114].

To circumvent some of the problems that arise with MeRIP-Seq and m^6^A-seq, m^6^A individual-nucleotide-resolution cross-linking and immunoprecipitation (miCLIP) was developed [96]. This method uses cross-linking–induced mutation site (CIMS) and cross-linking–induced truncation site (CITS) profiles generated during reverse transcription, due to the binding of specific antibodies at m^6^A residues and subsequent cross-linking by UV light, to identify precise positions of m^6^A-modified residues in RNA at single-nucleotide resolution. After next-generation sequencing and bioinformatical analysis of consensus motifs, identification of the modified residues is easier than in previously used methods.

Another very similar method, called photo-crosslinking-assisted m^6^A sequencing (PA-m^6^A-seq), was recently introduced [116]. PA-m^6^A-seq combines the incorporation of a photoactivatable ribonucleoside, 4-thiouridine (4-SU), into RNA and the immunoprecipitation with a m^6^A-specific antibody. By crosslinking the antibody to the introduced nucleotide and the subsequent transition of U/T to C during reverse transcription-polymerase chain reaction (RT-PCR), it is possible to narrow the resulting peaks after next generation sequencing to about 23 nt, which makes m^6^A position calling easier.

The m^6^A-level and isoform-characterization sequencing (m^6^A-LAIC-seq) uses a RNA immunoprecipitation protocol with m^6^A-specific antibodies and spike-in RNAs as internal standards coupled with whole-transcriptome sequencing to gain quantitative information about m^6^A modifications in poly(A)^+^ RNA fractions [93]. Using spike-in standards permits analysis of m^6^A levels per gene, but not the methylation stoichiometry of a single modified nucleotide.

One laborious, low-throughput method to confirm m^6^A sites at single-nucleotide resolution is site-specific cleavage and radioactive-labeling followed by ligation-assisted extraction and thin-layer chromatography (SCARLET) [88]. Moreover, it features the great advantage of quantifying the methylation status of a single modified nucleotide. The method relies on an induced, site-specific cut by RNase H followed by radioactive labeling of the resulting RNA fragments and a splint ligation to a single-stranded DNA oligo. After RNA digestion, gel purification, and Nuclease P1 treatment the radiolabeled mononucleotides are separated by thin layer chromatography and the methylation status can be analyzed quantitatively. Application of SCARLET is not limited to m^6^A, but can be applied to detect other RNA modifications as well, e.g., m^5^C and Ψ [87].

### 2.3. 5-Methylcytosine

5-methylcytosine (m^5^C) occurs in tRNA, rRNA, mRNA and lncRNA [72]. In mouse and human protein-coding transcripts, m^5^C sites are found about 100 nt downstream of the translation initiation site and in the UTRs [72,117,118]. Currently, two groups of m^5^C writers are known. The seven members of the NOP2/SUN RNA methyltransferase family member (NSUN) family, constituting the first group, methylate tRNA (NSUN2, NSUN6), rRNA (NSUN1, NSUN5), mRNA (NSUN2), ncRNA (NSUN2) as well as mt-rRNA (NSUN4) and mt-tRNA (NSUN3), respectively [64,66,67,68,69,119,120]. So far, NSUN7 substrates are obscure. However, mutations in the *Nsun7* gene lead to sperm motility defects, and therefore subfertility or complete infertility in male mice [70]. Moreover, mutations inside the *NSUN2* gene are linked with autosomal-recessive intellectual disability [121,122,123], and overexpression as well as increased copy numbers of *NSUN2* have been detected in human cancers [65,124,125].

The second writer protein group for m^5^C has only one member so far, namely, DNA methyltransferase-2 (DNMT2), which was previously thought to methylate DNA [126]. However, DNMT2 was found to act on tRNA with three tRNA substrates currently known [71,127]. DNMT2 expression levels, similar to other tRNA methyltransferases, were found to be frequently altered in cancer cells [128]. Indeed, data from hundreds of tumor samples collected by the COSMIC database reveal an overexpression of DNMT2 in several human cancers [129]. Additionally, more than 60 somatic mutations have been detected. An in vitro follow-up study examined 13 mutations and found varying results concerning DNMT2’s methylating activity [130]. However, translation of these data to pathways inside the cell’s regulatory network is difficult and needs to be addressed in context of the respective cancer type.

Importantly, a m^5^C eraser is still to be identified. Also, the functions of m^5^C are not well understood yet, although a recent study suggests a role for m^5^C in RNA transport [72]. The Aly/REF export factor (ALYREF), a mRNA export adaptor protein, was identified as a m^5^C binding (reader) protein, which promotes selective mRNA export from the nucleus [72].

Taken together, these findings suggest that m^5^C modifications in transcripts and the proteins involved in this pathway are important to control the fate and function of RNAs. A dysregulation of this system might contribute to pathophysiological states. Hence, a more detailed mapping of m^5^C modifications, as well as the discovery and functional analysis of m^5^C interacting proteins could contribute to a better understanding of the underlying molecular disease mechanisms.

#### m^5^C Detection

m^5^C is the most extensively studied base modification in DNA. Owing to the different chemical characteristics of DNA and RNA, the standard method of bisulfite treatment followed by sequencing had to be adapted to be used for m^5^C detection in RNA [131]. Bisulfite treatment leads to a chemical conversion of unmodified cytosine to uracil, whereas the methylated base remains unaltered. This difference can be detected by Sanger sequencing or after library construction by next-generation sequencing. Widespread m^5^C modifications could be detected by this method, called Bisulfite-seq, in tRNA, mRNA and ncRNA at single nucleotide resolution [118]. However, Bisulfite-seq has some drawbacks: cytosines in double-stranded RNA regions can remain unmodified by bisulfite treatment and are later falsely called m^5^C residues. Aside from the structure-related issue, sites with alternative modifications of cytosine can be misidentified as m^5^C sites, because those modified bases are usually resistant to bisulfite treatment as well. This is especially true for the closely related hm^5^C modification, which cannot be distinguished from m^5^C through Bisulfite-seq [132]. Therefore, candidate sites should be validated with complementary methods. For example, an alternative, immunoprecipitation-based protocol was recently developed [133]. Fragmented RNA is immunoprecipitated with a m^5^C-specific antibody or a control antibody, followed by library preparation and NGS. This protocol was applied to the RNA of the archaeon *Sulfolobus solfataricus,* which verified the Bisulfite-seq results [133].

Another, indirect m^5^C mapping method, called 5-Azacytidine–mediated RNA immunoprecipitation, or Aza-IP, takes advantage of the random incorporation of 5-Azacytosine into RNA during RNA synthesis inside the cell [127,134]. Overexpression of (epitope-tagged) RNA methyltransferase enzymes (RMTs) allows the immunoprecipitation of those enzymes with a (tag-) specific antibody. Importantly, 5-Azacytosine is a suicide substrate for m^5^C-RMTs due to the covalent link formed between the examined methyltransferase and its substrate RNA, which allows stringent washing steps. The Aza-IP is concluded by RNA fragmentation, cDNA library construction, and NGS. Comparison of resulting reads between the samples with a control or a specific antibody allows mapping of m^5^C sites. Additionally, the modified cytosine residue is read as a guanosine instead of cytosine during sequencing. This facilitates a precise calling of the candidate modified nucleotide. Identification of direct targets of DNMT2 and NSUN2 could be achieved with this method.

Indeed, NSUN2-specific methylation sites have been previously identified using yet another m^5^C detection method called methylation iCLIP (miCLIP) [120]. This method is derived from individual-nucleotide-resolution cross-linking and immunoprecipitation (iCLIP) [135], and abstains from chemical modifications of the RNA. To achieve this, a C271A mutant of NSUN2 was used, which forms a stable bond with its target cytosine residue due to the lack of its second cysteine in the catalytic center. The stable protein-RNA-complex was immunoprecipitated and NGS-based m^5^C mapping followed. A high cytosine appearance at position +1 in the cDNA libraries corresponds to the first nucleotide of the sequence reads, which means that reverse transcription terminated at the cross-link site of the cytosine with its modifying protein. New mRNA and ncRNA transcripts (e.g., vault RNAs) were identified as methylation targets, aside from confirming already known tRNA targets of NSUN2. Thus, identification of direct targets of NSUN2 can be achieved with single-nucleotide resolution using this protocol.

In summary, we are just at the beginning of a long journey to fully comprehend the breadth, dynamics and molecular functions of RNA modifications in mammalian cells. Recently developed high-throughput mapping approaches will enable us to characterize the epitranscriptome in diverse cellular contexts. Nevertheless, further technological improvements are needed to enhance the resolution and sensitivity of these methods. The discovery of additional writers, readers and erasers of the epitranscriptome, as well as a detailed analysis of already known ones, will spawn new research directions and might open the door for novel therapeutic strategies.

## 3. RNA Modifications in Cancer-Related lncRNAs

**Figure 2:**
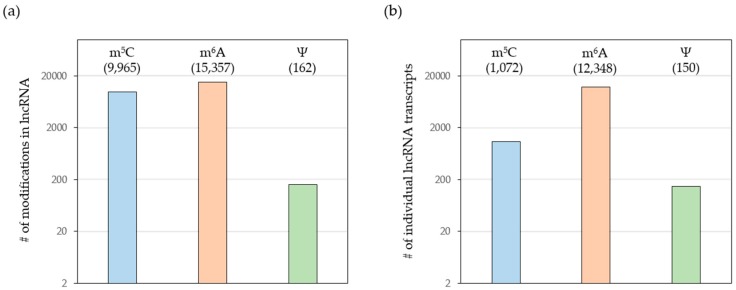
Integrated data analysis of three m^5^C, two m^6^A and three Ψ sequencing studies highlighting the total amount of modifications in lncRNAs (**a**) as well as the number of individual lncRNAs that contain the respective chemically modified nucleotide (**b**) (adapted from [132]).

Initial studies showed that RNA modifications have an impact on transcript localization, turnover and translation rates, thereby adding a new layer of gene expression control. However, most studies focused on mRNAs and much less is known about the functional relevance of RNA modifications in lncRNAs. Importantly, recent transcriptome-wide mapping studies revealed an overwhelming amount of RNA modifications in thousands of lncRNAs (Figure 2). Here, we will focus on a selection of lncRNAs with a well-established role in human cancers [20]. We will briefly introduce these lncRNAs and summarize our current knowledge about the RNA modifications previously identified in these non-coding transcripts.

### 3.1. MALAT1

Transcribed from chromosome 11 through RNA Pol II the metastasis associated lung adenocarcinoma transcript 1 (*MALAT1*), also known as *NEAT2* (nuclear-enriched abundant transcript 2), is a highly conserved and extremely abundant long non-coding RNA of ~8 kb in size that localizes to nuclear speckles [136]. Despite its ubiquitous expression in healthy organs, its genomic inactivation in mice is compatible with life and development [137,138,139].

Originally, *MALAT1* was identified in a subtractive hybridization screen for transcripts with an altered expression in stage I non-small cell lung cancers (NSCLCs) that either did or did not metastasize [140]. Follow-up studies on its cellular and molecular function in lung cancer established *MALAT1* as a master regulator of metastasis and a potential therapeutic target [141,142]. Furthermore, *MALAT1* has been found to control proliferation, migration and apoptosis in many different human cancers, e.g., pancreatic cancer, hepatoma and ovarian cancer [143,144,145,146]. Furthermore, its overexpression can increase drug resistance as shown for temozolomide in glioblastoma cells [147].

Mechanistically, *MALAT1* is thought to fulfill its cellular functions by regulating gene expression levels as well as alternative splicing [142,143,148,149]. Interestingly, Wilusz et al. showed that *MALAT1* undergoes a maturation process that yields a mature and stable transcript [150]. Here, the 3′-terminus of the *MALAT1* is cleaved by RNase P at position A6690 that follows after an adenosine-rich tract. This produces two ncRNAs: a long, 5′-capped *MALAT1* transcript with a short poly(A)-tail like moiety and a small, tRNA-like ncRNA, the so called *MALAT1*-associated small cytoplasmic RNA (mascRNA) [150]. While the *MALAT1* transcript remains in the nucleus, the mature 61-nt mascRNA is exported to the cytoplasm where it might act as an immune regulator [151]. Importantly, processed *MALAT1* transcripts contain a 3′-triple-helical RNA stability element consisting of a U-rich internal loop that associates with a downstream A-rich tract to protect the *MALAT1* transcript from degradation. This triple helix is recognized and bound by the m^6^A writer METTL16 [152]. This raises the possibility of a m^6^A modification being present in this triple-helix. Alternatively, *MALAT1* could serve a role as a regulator of RNA processing or modification events through guiding METTL16 onto its RNA targets.

It has recently been shown that *MALAT1* can carry m^6^A modifications [88]. The authors used SCARLET to determine the m^6^A status of *MALAT1* in different cell lines focusing on the largest m^6^A/MeRIP-Seq peak previously identified [57,97]. This peak contains seven predicted m^6^A-consensus motifs (RRACH), and four of these consistently carried methylated residues across the four different cell-types tested. However, the modification rate varied: two positions (A2515 and A2577) displayed the highest (41–67% and 51–88%, respectively) modification rate, followed by A2611 (13–49%) and A2720 (7–14%). Only a small fraction of *MALAT1* molecules (2–3%) carried the m^6^A modification at the other predicted sites (A2674/2684/2698) in two out of four cell lines. Importantly, secondary structure prediction and mapping experiments demonstrated that the two residues with the highest m^6^A rate (i.e., A2515 and A2577) are located in hairpin stems. Subsequent structural mapping assays using methylated and unmethylated synthetic RNA oligonucleotides in conjunction with a set of structure-sensitive nucleases revealed that the presence of m^6^A in the hairpin stem increases the opening of the stem, i.e., reduces duplex stability [88]. In a later study, the authors could show that adenosine methylation at position A2577 destabilizes the hairpin stem, making the opposing U-tract more single-stranded and accessible for RNA-binding proteins, e.g., hnRNP C [61]. Furthermore, nuclear magnetic resonance and Förster resonance energy transfer studies demonstrated that the overall structure of the *MALAT1* hairpin is maintained upon m^6^A modification, but the nucleobases of the hairpin stem are more flexible and solvent accessible [153]. These results support a model in which m^6^A regulates protein binding through its influence on RNA structure (“m^6^A switch”) [61]. While the *MALAT1* hairpin stem is the first example of such an m^6^A-switch, changes induced by m^6^A modifications might apply to a much larger family of m^6^A-regulated RNA structures. Of note, modification of *MALAT1* with m^6^A is highly dynamic and can be modulated by heat shock, UV and growth factor treatments in HepG2 cells [57]. Thus, it would be interesting to learn more about the functional significance of these conditional modifications.

Next to m^6^A, *MALAT1* also contains several pseudouridine residues at positions U5160, U5590 and U3374 [77,87]. However, their impact on *MALAT1* structure, protein interaction or molecular function are not known.

Additionally, Squires et al. identified several putative m^5^C sites within *MALAT1* through RNA bisulfite conversion combined with NGS [118]. However, the enzymes responsible for the m^5^C modification of *MALAT1* are unknown, but DNMT2 and NSUN2 could potentially be excluded, since *MALAT1* was only slightly enriched after respective Aza-IPs in HeLa cells [127]. Hence, other m^5^C writers should be tested in the future.

### 3.2. HOTAIR

The Hox transcript antisense intergenic RNA (*HOTAIR*) is a long, intergenic ncRNA of ~2.2 kb that is transcribed from the antisense strand of the developmental *HOXC* gene cluster on chromosome 12 [154]. Dysregulated expression of *HOTAIR*, which promotes metastasis in several cancer types, is often found in human cancers, e.g., melanoma, breast, hepatocellular, gastric, colorectal or pancreatic carcinoma, and its expression is correlated with poor prognosis, e.g., in colorectal cancers [155,156,157,158,159,160]. Moreover, a recent study showed that *HOTAIR* can serve as a plasma-derived biomarker for the diagnosis and monitoring of NSCLC [161].

Mechanistically, *HOTAIR* is located in the nucleus and the characterization of the molecular interactions of this *trans*-acting ncRNA revealed two regions involved in direct interactions with chromatin-modifying complexes [162]. One interaction site is located in a ~300 nt region at the 5′-end, enabling the direct binding to the polycomb-repressive complex 2 (PRC2), a complex displaying histone methyltransferase activity. The interaction with *HOTAIR* is required for PRC2 occupancy and histone H3 lysine-27 trimethylation (H3K27me3) resulting in inhibition of gene expression across 40 kb of the *HOXD* gene locus [154,162]. The second, ~700 nt long interaction site, is located at the 3′-end of *HOTAIR* and is required for the interaction with the histone demethylase complex lysine specific demethylase 1 (LSD1)/co-repressor of RE1-silencing transcription factor (CoREST)/RE1 silencing transcription factor (REST) [162]. The ability of *HOTAIR* to tether these two distinct chromatin-modifying complexes enables coupled histone H3K27 methylation and lysine 4 demethylation (H3K4) to induce epigenetic gene silencing.

Interestingly, a previous study identified a specific cytosine methylation in *HOTAIR* at position C1683 occurring with complete penetrance (i.e., 100% modification rate) and present in all five cell lines tested [163]. However, Aza-IPs of DNMT2 and NSUN2 did not enrich *HOTAIR* suggesting that other methyltransferases might be responsible for this modification [127]. Importantly, since the methylated cytosine residue is located within the 700 nt LSD1 binding motif, it is tempting to speculate about a regulatory impact of the epitranscriptome on the epigenome. However, a methylation-dependent interaction between *HOTAIR* and LSD1 with downstream effects on histone H3 lysine 4 methylation changes has yet to be shown.

While additional chemical modification in *HOTAIR* have not been analyzed in more detail so far, Meyer et al. identified a single m^6^A peak region (126 nt) in the first half of *HOTAIR*, not overlapping with m^5^C, in HEK293T cells using MeRIP-Seq [97]. In contrast, Dominissini et al. did not find any m^6^A signal in *HOTAIR* using HepG2 cells or human brain tissue despite the presence of several DRACH consensus motifs [57].

Studies form Carlile et al. using Pseudo-Seq in HeLa cells, and Li et al. applying CeU-Seq in HEK293T cells, could not establish *HOTAIR* as a target for pseudouridylation [77,87]. However, additional cell systems and tissues should be analyzed to obtain a more comprehensive view about the chemical modifications and their putative functions in *HOTAIR*.

### 3.3. XIST

The process of X inactivation, i.e., the transcriptional silencing of one of the pair of X chromosomes, is initiated early in female mammalian development to provide dosage equivalence between males and females. *XIST* is a ~17 kb lncRNA that is expressed from a region called X inactivation center (XIC). *XIST* is essential for the initiation and spread of X-inactivation by coating the chromosome in *cis* [164,165,166,167]. Recently, three independent studies mapped the *XIST* RNA-protein interactome thereby providing further insights into the molecular mechanisms of *XIST*-mediated heterochromatinization [168,169,170,171]. Despite the use of distinct methodologies and different cellular systems, several overlapping proteins were identified in these studies including the previously described interactor hnRNP U as well as the newly identified binders SPEN and RBM15 [168,170,171,172]. However, the functional relevance of these interactions needs to be assessed in more detail.

In line with this, a recent study revealed a RBM15/METTL3/YTHDC1 pathway of m^6^A formation and recognition that is required for *XIST*-mediated transcriptional repression [54]. In detail, the authors could show that the high m^6^A modification rate (78 m^6^A residues) of *XIST* is dependent on RBM15 and its paralogue RBM15B, two RNA-binding proteins that link the m^6^A methylation complex to *XIST* through interaction with WTAP that in turn binds to the methyltransferase METTL3. Finally, m^6^A residues in *XIST* are recognized by YTHDC1 which leads to gene silencing. How exactly YTHDC1 binding to *XIST* leads to gene silencing remains unclear, but might involve additional molecular interactions between YTHDC1 and other proteins with well-established roles in the initiation of transcriptional silencing [54].

In addition to m^6^A, *XIST* was also shown to contain methylated cytosine residues [163]. A 5’-region of *XIST*, termed repeat A-region, consists of 8.5 repeats with 26 nt per full repeat and is required for the association with PRC2 [167]. Characterization of posttranscriptional chemical modifications in *XIST* revealed five methylated cytosines within repeat 8: C701, C702, C703, C711 and C712. The methylation rate of individual cytosine residues was between 19–24%, and a simultaneous modification of all five residues was detected in 19% of the sequences analyzed. Interestingly, non-methylated, but not methylated RNA oligonucleotides spanning the R8 tetra-loop and part of the inter-repeat helix of *XIST* were bound by PRC2 indicating that m^5^C, in contrast to m^6^A, can prevent *XIST*-protein interactions. However, no m^5^C modification was detected at the corresponding cytosines C668, 669, 670 and 678 in the A-region of mouse *Xist,* arguing against a conserved mechanism [163].

Finally, a third chemical modification in *XIST*, a pseudouridine residue at position U11249, was recently discovered [87]. However, the functional role of this modification is currently unknown.

Interestingly, X chromosome aneuploidies have long been associated with human cancers, but causality has not been established. A recent study in mice made a step forward. Here, deletion of *Xist* in the blood compartment of mice led to the development of a highly aggressive myeloproliferative neoplasm and myelodysplastic syndrome (mixed MPN/MDS) with 100% penetrance establishing a tumor-suppressive role of *Xist* [173]. Intriguingly, MDS is more common in women and *XIST* deletions and X chromosome duplications have been found in MPN, MDS, and myeloid cancers [174,175,176,177,178]. However, the association is not restricted to women, because extra X chromosomes are seen in acute lymphoblastic leukemias (ALL), AML, acute nonlymphoblastic leukemia (ANLL), adult T cell leukemia, chronic myeloid leukemia (CML), erythroleukemia and non-Hodgkin lymphoma of both sexes and ~60% of childhood ALL display extra X chromosomes and an extra X may be the only aneuploidy in some CML [173,179,180]. In contrast to these hematological cancers, *XIST* gene copy number amplifications and increased expression levels have been detected in other cancers, e.g., microsatellite-unstable colorectal carcinoma (CRC) [181]. Elevated expression of *XIST* was recently associated with poor survival in CRC patients, and knockdown of *XIST* inhibited proliferation, invasion, epithelial-mesenchymal transition (EMT) and CRC stem cell formation in vitro, as well as tumor growth and metastasis in vivo [182].

Hence, *XIST* might have context-dependent pro- or antitumor functions in human cancers and it would be interesting to know, if chemical modifications in *XIST* can shift the balance in one or the other direction.

### 3.4. SRA1

The steroid receptor RNA activator (*SRA*) is an example of a bifunctional gene that is active as a lncRNA (*SRA1*), yet also encoding a conserved protein (SRAP) [183]. *SRA1* has a large number of isoforms, some of which display tissue-specific expression [184,185]. While most of the isoforms share a central core region that is necessary for its function as a coactivator, only some isoforms contain an open reading frame for SRAP production [183,186,187]. Both, the coding and the non-coding part of *SRA* have been described to be involved in the regulation of the transcriptional activity of different hormone receptors (androgen receptor, estrogen receptor, glucocorticoid receptor, thyroid hormone receptor, and retinoic acid receptor) in a cell-specific manner indicating potential anti-cancer targets [186,188]. However, the role of *SRA1* in carcinogenesis is not fully understood yet. For example, transgenic overexpression of *SRA1* in mice caused hyperplasia and morphological abnormalities in steroid hormone responsive tissues, but did not induce tumors and was accompanied by higher apoptosis rates. *SRA1* also antagonized Ras-induced tumor formation [184].

Interestingly, the pseudouridine synthase Pus1 was previously identified as an interaction partner and coactivator of retinoic acid receptors (RARs), as well as other class I and II nuclear receptors in mouse cells [40]. Furthermore, Pus1 was shown to bind and modify *SRA1*, which is required for its role as a coactivator. In a subsequent study, the same authors identified a specific uridine residue in *SRA1* (U206) whose modification by Pus1 (or Pus3) might induce a functional switch to allow *SRA1* to act as coactivator or corepressor [189]. This could partially explain cell-type specific functions of *SRA1*.

Other chemical modifications of *SRA1* have not been described or functionally analyzed so far. However, a close examination of transcriptome-wide m^6^A datasets warrants further investigations to clarify a putative link between the epitranscriptome and *SRA1*-dependent nuclear receptor signaling events [57,97].

### 3.5. Additional lncRNAs with Posttranscriptional Chemical Modifications

Mining published datasets for lncRNAs reveals a broad selection of chemically modified transcripts (Table 2). For example, Dominissini et al. mapped m^6^A to well-known lncRNAs, e.g., *PVT1* and *NEAT1* as well as uncharacterized lncRNA transcripts [57]. Having a closer look at m^5^C sites in lncRNAs, Squires et al. identified several putative target sites, e.g., in *SNHG12*, *GAS5*, *TERC*, *RPPH1* and *ANRIL* [118]. However, only a few studies exist that have carefully mapped the position of modified residues in single transcripts, e.g., m^6^A in the lncRNA taurine up-regulated 1 (*TUG1*) (A1114) [88]. The same is true for pseudouridine residues in lncRNAs. Transcriptome-wide studies identified pseudouridine sites, e.g., in *LRRC75A* antisense *RNA 1* (*LRRC75C-AS1*; U1537) and small nucleolar *RNA* Host Gene 1 (*SNHG1*; U1766) [77]. Individual studies focusing on specific lncRNAs mapped a pseudouridine at position U250 in *RN7SK* and Hussain et al. could identify *RN7SK* as a target for the m^5^C-introducing enzyme NSUN2 in HEK293T cells [77,120]. Kcnq1ot1, an imprinted lncRNA interacting with G9a and PcG proteins with elevated levels in patients with myocardial infarction and a function in transcriptional interference, contains a pseudouridine at position U64919 [87]. In addition, Li et al. also identified a heat shock-inducible pseudouridine (U19886) in Kcnq1ot1 as well as several additional pseudouridine sites within different lncRNAs, e.g., *ST7-AS1* (U1138), *ZFAS1* (U569), *SNHG7* (U292), *DICER1-AS1* (U463), including also many inducible sites, e.g., *DLEU2L* (U1379, H_2_O_2_-inducible), *APTR* (U1282, H_2_O_2_-inducible), or *MAGI2-AS3* (U3659, heat shock-inducible) [87]. The transcriptome-wide mapping of pseudouridine in HEK293T cells by Schwartz et al. revealed a highly conserved position (U307) as well as a putative site at position U179 in the telomerase RNA component (*TERC*) [85].

Nevertheless, the relevance of these epitranscriptomic changes in lncRNAs are largely unknown and require additional validation, as well as functional follow-up studies.

## 4. Conclusions

The research field of epitranscriptomics has made huge strides in the last few years. High-throughput sequencing techniques enabled nearly transcriptome-wide modification detection and generated enormous amounts of data [191]. Validation of this data avalanche is tedious and more difficult than it seems on first sight, and the majority of modification sites should be treated as candidate sites [192]. New approaches and techniques are needed to validate modification data and to rush the field forward. Third-generation sequencing technology, improved chromatography methods and newly devised mass spectrometry protocols look promising to help gain new insights into the epitranscriptome landscape [193,194]. Information about the stoichiometry of each modified site will be needed to fully understand the importance of RNA modifications and their contribution to the highly dynamic cellular processes. Newly discovered binding proteins, be it writers, readers or erasers, will provide hints about which pathways are influenced or directed by RNA modifications, and will broaden our understanding of post-transcriptional regulatory mechanisms.

**Figure 3:**
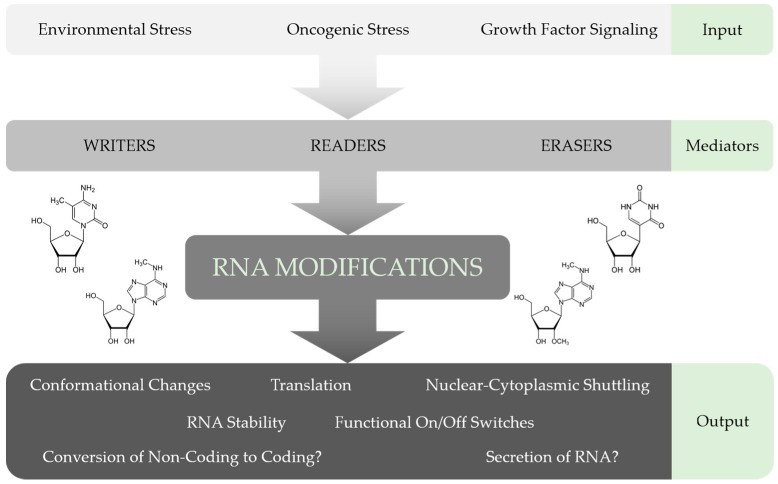
Putative information flow impacting chemical RNA modifications. Internal and external signals lead to epitranscriptomic changes, which are applied by writer and eraser proteins, and subsequently conveyed by reader proteins. Some functions of these epitranscriptomic marks have already been shown while additional mechanisms can be envisioned.

Furthermore, investigating the distribution and function of chemical modifications in lncRNAs, as well as their association with the relevant proteins in more detail, will contribute towards an integrative understanding of the multilayered gene expression control mechanisms active in mammalian cells (Figure 3). Intriguingly, some lncRNAs seem to have cell-type specific functions. For example, *MALAT1* was shown to be important for cell proliferation, apoptosis or motility by regulating alternative splicing and gene expression in one cell system, but affecting the expression of different genes in other cell lines [143]. Hence, it would be interesting to study the impact of chemical modifications on the cell-type-specific functions of *MALAT1* and other lncRNAs.

Moreover, recent large-scale ribosome footprinting studies have made the surprising and controversial observation that lncRNAs interact with ribosomes and suggest that lncRNAs are capable of translating short peptides [195,196,197,198,199,200]. However, others have reached different conclusions [201]. Interestingly, a recent study suggests that ribosomes are the default destination for the majority of cytoplasmic lncRNAs and they may also play a role in lncRNA turnover [202]. Given the previously described ability of pseudouridine to expand the genetic code [76], it is tempting to speculate about epitranscriptome-based mechanisms that regulate ribosome-bound transcript degradation or might convert non-coding RNAs into coding ones.

In summary, we are only starting to unravel the full breadth of the transcriptome, which comes in many (chemical) flavors. To date, more than 150 modifications have been identified in RNA, but only a handful can be currently mapped with high-throughput methods. This offers plenty of opportunities to discover novel regulatory principles. Moreover, proteins involved in the epitranscriptomic cascade might represent interesting therapeutic targets. However, our current knowledge about the epitranscriptomic changes that might occur during carcinogenesis, as well as their functional relevance on single molecule level, especially in lncRNAs, is still very limited. Hence, further molecular and mechanistic investigations are needed. These studies might pave the way for the development of novel therapeutics and might help to identify biomarkers for early cancer detection and therapy response.

## Figures and Tables

**Table 1:** Known writer, reader, and eraser proteins for chemical RNA modifications.

RNA Modification	Writer	Reader	Eraser
Ψ	PUS1 [39,40] PUSL1 [41] PUS3 [42,43] PUS7 [44] PUS7L [41] PUS10 [45] RPUSD1 [41] RPUSD2 [41] RPUSD3 [46] RPUSD4 [46] TRUB1 [44] TRUB2 [46] DKC1 [47]		
m^6^A	METTL3 [48] METTL14 [49,50,51] WTAP [49,50,51,52] KIAA1429 [51,53] RBM15 [53,54] RBM15B [54] METTL16 [55]	YTHDF1 [56] YTHDF2 [57] YTHDF3 [57] YTHDC1 [58] YTHDC2 [54] eIF3 [59] HNRNPA2B1 [60] HNRNPC [61]	ALKBH5 [62] FTO [63]
m^5^C	NSUN1 [64] NSUN2 [65] NSUN3 [66] NSUN4 [67] NSUN5 [68] NSUN6 [69] NSUN7 [70] DNMT2 [71]	ALYREF [72]	

**Table 2:** Selected lncRNAs and their recently identified chemical modifications.

lncRNA	Modification	No. of Modified Residues	Reference
*ANRIL*	m^6^A	1	[97]
	m^5^C	2	[118]
*DICER1-AS1*	m^6^A	2	[97]
	Ψ	1	[87]
*GAS5*	m^5^C	2	[118]
*HOTAIR*	m^6^A	1	[97]
	m^5^C	1	[163]
*Kcnq1ot1*	Ψ	1	[87]
*LRRC75A-AS1*	Ψ	1	[77]
*MALAT1*	m^6^A	3	[57]
		3	[97]
	m^5^C	7	[118]
	Ψ	3	[77]
		3	[87]
*NEAT1*	m^6^A	1	[57]
	m^5^C	7	[118]
*PVT1*	m^6^A	2	[57]
		1	[97]
	m^5^C	1	[118]
		1	[120]
	Ψ	1	[77]
*RPPH1*	m^5^C	4	[118]
		1	[127]
		1	[120]
*SNHG1*	Ψ	1	[77]
*SNHG7*	Ψ	1	[87]
*SNHG12*	m^5^C	2	[118]
*SRA1*	m^6^A	1	[57]
		4	[97]
	Ψ	1	[40,189]
*ST7-AS1*	Ψ	1	[87]
*TERC*	m^5^C	3	[118]
	Ψ	2	[85]
		6	[190]
*TUG1*	m^6^A	1	[88]
		11	[97]
*XIST*	m^6^A	1	[57]
		14	[97]
	m^5^C	5	[163]
	Ψ	1	[87]
*ZFAS1*	Ψ	1	[85]

## Abbreviations

4-SU	4-thiouridine
ALKBH5	α-ketoglutarate-dependent dioxygenase alkB homolog 5
ALL	Acute lymphoblastic leukemia
ALYREF	Aly/REF export factor
AML	Acute myeloid leukemia
ANLL	Acute nonlymphoblastic leukemia
ANRIL	Antisense Non-coding RNA in the *INK4* Locus
APTR	Alu-mediated CDKN1A/p21 transcriptional regulator
ARE	AU-rich element
Aza-IP	5-azacytidine-mediated RNA immunoprecipitation
CeU-Seq	N3-CMC-enriched pseudouridine sequencing
CIMS	Cross-linking–induced mutation site
CITS	Cross-linking–induced truncation site
CMCT	*N*-cyclohexyl-*N*′-(2-morpholinoethyl)carbodiimide metho-p-toluenesulfonate
CML	Chronic myeloid leukemia
CoREST	Co-repressor of RE1-silencing transcription factor
CRC	Colorectal cancer
DICER1-AS1	DICER1 antisense RNA 1
DKC1	Dyskerin pseudouridine synthase 1
DLEU2L	Deleted in lymphocytic leukemia 2-like
DNMT2	DNA methyltransferase-2
eIF3	Eukaryotic initiation factor 3
eIF4E	Eukaryotic initiation factor 4E
EMT	Epithelial-mesenchymal transition
FTO	Fat mass- and obesity-associated protein
GAS5	Growth arrest specific 5
GBM	Glioblastoma
GSC	Glioblastoma stem-like cells
H3K27	Histone H3 lysine-27
H3K4	Histone H3 lysine-4
hnRNP U	Heterogeneous nuclear ribonucleoprotein U
HNRNPA2B1	Heterogeneous nuclear ribonucleoprotein A2/B1
HNRNPC	Heterogeneous nuclear ribonucleoprotein C
HOTAIR	HOX antisense intergenic RNA
HOXC/D	Homeobox C/D
HuR	Human antigen R
iCLIP	Individual-nucleotide-resolution crosslinking and immunoprecipitation
lncRNA	Long non-coding RNA
LRRC75C-AS1	LRRC75A antisense RNA1
LSD1	Lysine specific demethylase 1A
m^5^C	5-methylcytosine
m^6^A	*N*^6^-methyladenosine
m^6^A_m_	*N*^6^,2′-*O*-dimethyladenosine
MAGI2-AS3	MAGI2 antisense RNA 3
MALAT1	Metastasis Associated Lung Adenocarcinoma Transcript 1
mascRNA	MALAT1-associated small cytoplasmic RNA
MAT2A	Methionine adenosyltransferase 2A
MDS	Myelodysplastic syndrome
MeRIP-Seq	M^6^A specific methylated RNA immunoprecipitation-sequencing
METTL	Methyltransferase-like protein
miRNA	MicroRNA
MPN	Myeloproliferative neoplasm
mRNA	Messenger RNA
MS	Mass spectrometry
mtRNA	Mitochondrial RNA
ncRNA	Non-coding RNA
NEAT1	Nuclear paraspeckle assembly transcript 1
NEAT2	Nuclear-Enriched Abundant Transcript 2
NGS	Next-generation sequencing
NSCLC	Non-small cell lung cancer
NSUN	NOP2/Sun RNA methyltransferase family member
nt	Nucleotide
PA-m^6^A-seq	Photo-crosslinking-assisted m^6^A sequencing
PcG	Polycomb group
piRNA	PIWI-interacting RNA
PRC2	Polycomb-repressive complex 2
PTM	Posttranslational modification
PUS	Pseudouridine synthase
PUS7L	Pseudouridylate synthase 7 like
PUSL1	Pseudouridylate synthase-like 1
PVT1	Pvt1 oncogene
RAR	Retinoic acid receptor
RBP	RNA-binding protein
REST	RE1-silencing transcription factor
RBM15	RNA-binding motif protein 15
RMT	RNA methyltransferase
RN7SK	RNA 7SK small nuclear
RPPH1	Ribonuclease P RNA component H1
RPUSD	RNA pseudouridylate synthase domain containing
rRNA	Ribosomal RNA
RT-PCR	Reverse transcription-polymerase chain reaction
SAM	*S*-Adenosyl methionine
SCARLET	Site-specific cleavage and radioactive-labeling followed by ligation-assisted extraction and thin-layer chromatography
siRNA	Small interfering RNA
SNHG	Small nucleolar RNA host gene
snoRNA	Small nucleolar RNA
snRNA	Small nuclear RNA
SPEN	Spen family transcriptional repressor
SRA	Steroid receptor RNA activator
SRAP	Steroid receptor RNA activator protein
ST7-AS1	ST7 antisense RNA 1
TERC	Telomerase RNA component
tRNA	Transfer RNA
TRUB	TruB pseudouridine synthase family member
TUG1	Taurine up-regulated 1
UTR	Untranslated region
VIRMA	Vir like m^6^A methyltransferase associated
WTAP	Wilms’ tumor 1-associating protein
XIC	X inactivation center
XIST	X-inactive specific transcript
YTH	YT521-B homology
YTHDC	YTH domain-containing protein
YTHDF	YT521-B homology domain family
ZFAS1	ZNFX1 antisense RNA 1
Ψ	Pseudouridine
